# PNCK depletion inhibits proliferation and induces apoptosis of human nasopharyngeal carcinoma cells *in vitro* and *in vivo*

**DOI:** 10.7150/jca.33698

**Published:** 2019-12-03

**Authors:** Yuanji Xu, Jiling Wang, Shaoli Cai, Guanghao Chen, Nanyang Xiao, Yajuan Fu, Qi Chen, Sufang Qiu

**Affiliations:** 1 Department of Radiation Oncology, Fujian Medical University Cancer Hospital & Fujian Cancer Hospital, Fuzhou, China.; 2 Department of Medical Oncology, The First Hospital of Putian City, Putian, China.; 3 Biomedical Research Center of South China, Fujian Normal University, Fuzhou, China.; 4 The Key Laboratories of Innate Immune Biology of Fujian Province, Fuzhou, China.; 5 Longyan People Hospital, Longyan, China.; 6 Fujian Provincial Key Laboratory of Translational Cancer Medicine, Fuzhou, China.

**Keywords:** NPC, PNCK, gene expression profiling.

## Abstract

**Purpose:** Recent studies indicate that pregnancy upregulated non-ubiquitous calmodulin kinase (PNCK) is significantly up-regulated in breast and renal carcinomas. However, the expression profile and its biological relevance of PNCK in nasopharyngeal carcinoma (NPC) have not been elucidated.

**Methods:** The expression level of PNCK was detected in specimens of NPC (n=10) and normal tissues (n=10) by real-time PCR and immunohistochemistry. Celigo Cell Counting and MTT assay were used to measure cell viability. Apoptosis was detected by flow cytometric analysis and caspases 3/7 activity assay. Real-time PCR and Western blotting were performed to evaluate the expression of PNCK. The bioluminescence imaging was used to evaluate the effects of PNCK knockdown on tumor growth using a xenograft animal model. The global gene expression profile was determined in wild type and PNCK-depleted CNE-2 cells via transcriptomics analysis. For mechanical investigation, the changes of PI3K/AKT/mTOR signaling pathway were detected by Western blotting.

**Results:** The mRNA and protein levels of PNCK were increased in human NPC samples. *In vitro* experiments showed that shRNA or CRISPR-Cas9 mediated silencing of PNCK inhibited proliferation and induced apoptosis in NPC cells. In addition, *in vivo* assay revealed that knockdown of PNCK suppressed tumor growth. Consistently, a significant reduction of tumor bioluminescence in mice inoculated with PNCK-knockdown cells compared to that of control cells. In gene expression, the transcriptomics analysis revealed that there were 589 upregulated genes and 589 downregulated genes in PNCK-knockdown cells. Ingenuity Pathway Analysis (IPA) identified significant changes of PI3K/AKT/mTOR signaling pathway in PNCK-knockdown cells. Furthermore, western blot analysis revealed that interference with PNCK reduced the phosphorylation levels of PI3K, AKT and mTOR in CNE-2 cells.

**Conclusion:** This study for the first time demonstrates that knockdown of PNCK could suppress growth and induce apoptosis of NPC cells both *in vitro* and *in vivo* by regulating PI3K/AKT/mTOR signaling pathway*.* These findings suggest that PNCK might be a novel therapeutic target for NPC treatment.

## Introduction

Nasopharyngeal carcinoma (NPC) is a squamous epithelial malignant tumor arising from the lateral wall surface of nasopharynx with a high incidence in southern China, Japan and Southeast Asia 1. Prevalence survey shows that the southern Chinese have the highest incidences of NPC in the world 2. Genetic, environmental and microbial factors are thought to be involved in the carcinogenesis of NPC 3. Due to the high sensitivity of NPC cells to ionizing radiation, radiotherapy is the main modality for treatment of NPC. However, some NPC patients have a poor prognosis because of no suitable biomarker for early detection and poor understanding of the molecular mechanisms 4-6. Thus, the further understanding of the underlying mechanisms of NPC tumorigenesis will contribute to develop novel options for the diagnosis and therapy of NPC.

Calmodulin (CaM) kinases are a group of serine/threonine kinases that catalyze various biological processes, such as neurotransmitter release, muscle contraction, proliferation, differentiation, and apoptosis 7-9. Pregnancy-upregulated nonubiquitous calmodulin kinase (PNCK) is a novel member of the CaM kinase family, which shares 45-70% sequence similarity with the kinase domain of the CaM kinase family. PNCK is widely expressed in the central nervous system and in a variety of tissues including heart, breast, brain, uterus, and stomach 10. Recent studies have found that PNCK play critical roles in cytoplasmic and nuclear signal transduction, thereby regulating various biological processes 11,12. However, the molecular mechanism by which PNCK contributes to the malignant NPC remains largely unknown.

Therefore, it is critical to elucidate the mechanism by which PNCK exerts its functional role in the progression of NPC. In the present study, for the first time, we explored the functional relevance of PNCK using NPC tissue samples, cell lines and animal models. For mechanical investigation, transcriptome analysis was performed and many signaling pathways were found to be altered. Analysis of clinical samples revealed a significant elevation of PNCK in NPC. Moreover, *in vitro* and *in vivo* studies showed that knockdown of PNCK substantially inhibited growth and induced apoptosis in human NPC cells. In addition, transcriptomic analysis revealed that PI3K/AKT/mTOR pathway was remarkably changed, which may be responsible for PNCK-mediated cellular behaviors. Taken together, our study indicates that the PNCK could be a target for treatment of NPC.

## Materials and methods

### Patients and tissue specimens

In this study, 8 freshly frozen NPC and 10 normal nasopharyngeal tissue were collected in Fujian Cancer Hospital between January 2017 and March 2017. Then, paraffin-embedded specimens of NPC (n=10) and normal tissues (n=10) were used for gene expression analysis. These patients had no radiotherapy or chemotherapy history before biopsy. NPC was pathologically confirmed by two senior pathologists who were blinded to the clinical information of patients. This study was approved by the Institute Research Medical Ethics Committee of Fujian Cancer Hospital, Fujian Medical University Cancer Hospital (#2017-051-01), with a written consent form signed by patients.

### Cell culture

The human NPC cell lines (CNE-2, CNE-1 and 5-8F) were purchased from the Cell Resource Center (Shanghai Institutes for Biological Sciences, China Academy of Sciences). NPC C666-1 cell line was a gift form Prof. Geoge S.W. Tsao of the University of Hong Kong. Cells were cultured in Dulbecco's Modified Eagle Medium supplemented with 10% FBS, 100 U/mL penicillin, and 100 U/mL streptomycin, and were maintained at 37°C in 5% CO_2_ incubator.

### Transcriptome analysis

Total RNAs were extracted using TRIZOL Reagent (Life technologies, Carlsbad, CA, USA) following the manufacturer's instructions and checked for RNA integrity by an Agilent Bioanalyzer 2100 (Agilent technology, Santa Clara, CA, USA). Qualified total RNA was further purified by RNeasy microkit (QIAGEN, GmBH, Germany) and RNase Free DNase Set (QIAGEN, GmBH, Germany). Total RNAs were amplified, labeled and purified by using GeneChip 3'IVT Express Kit (Affymetrix, Santa Clara, CA, USA) followed the manufacturer's instructions to obtain biotin labeled RNA. Array hybridization and wash was performed using GeneChip® Hybridization, wash and stain Kit (Affymetrix, Santa Clara, CA) in Hybridization Oven 645 (Affymetrix, Santa Clara, CA) and Fluidics Station 450 (Affymetrix, Santa Clara, CA) followed the manufacturer's instructions. Slides were scanned by GeneChip® Scanner 3000 (Affymetrix, Santa Clara, CA, US) and Command Console Software 3.1 (Affymetrix, Santa Clara, CA, US) with default settings. Differentially expressed genes with statistical significance, a fold change filtering between two samples was performed and the default threshold was ≥1.5 fold-change. The biological processes were identified using Ingenuity Pathway Analysis (http://www.ingenuity.com/products/ipa).

### Cell proliferation assay

Cell proliferation was determined using MTT [3-(4, 5-dimethylthiazol-2-yl)-2, 5-diphenyl tetrazolium bromide] assay (Roche Diagnosis). Briefly, cells were plated into 96-well plates at the density of 2,000 cells/well in triplicates and cultured in DMEM supplemented with 10% FBS. After 24 h incubation at 37˚C, 20 μl of 5mg/ml MTT was added and further cultured for 4 hours. After discard of culture media, 100 μl/well of dimethyl sulfoxide was added to well and the optical density was measured at 490 nm using a Microplate Reader (Bio-Rad, Hercules, CA, USA). All experiments were performed at least three times.

### Apoptosis assay

Cells were collected by trypsinization, washed twice with PBS and fixed in 80% ice-cold ethanol in PBS. Then, cells (≥5×10^5^) were re-suspended in 200 μl binding buffer and incubated with 10 μl staining solution containing FITC-conjugated annexin V antibody (cat: 88-8007, eBioscience, CA, USA). After incubation for 15 min at room temperature in the dark the apoptotic cells were detected by flow cytometer following the manufacturer's instruction.

### Caspase 3/7 activity assay

The Caspase 3/7 activity was determined in each group using a commercial kit (Caspase-Glo® 3/7 Assay, Promega, Shanghai) according to the manufacturer's instruction. In brief, 100μl Caspase3/7 reagent was added to each well of the plates and incubated at room temperature for 2h. Absorbance values were detected using a microplate reader at A405nm (Tecan infinite, Mannedorf, Switzerland).

### Immunohistochemical staining

The paraffin-embedded blocks were cut into 3-μm thickness sections and mounted on glass slides coated with poly-L-lysine, deparaffinized with xylene and rehydrated with gradient ethanol. The slides were heated and incubated in a 10 mM citrate buffer. After inactivating endogenous peroxidase by H_2_O_2_, the slides were incubated with primary antibody targeting PNCK (1:20, HPA007458, Sigma, USA) for 1 h followed by incubation with biotinylated secondary antibody. The staining intensity was considered as negative (0), weak (1), moderate (2), or strong (3). The percentage of positive staining was scored as 0% (0), <25% (1), 25-50% (2), 50-75% (3), or >75% (4). The immunostainings of PNCK was evaluated by two pathologists without knowledge of patient characteristics and a consensus was provided on staining patterns.

### Real time PCR

Total RNA from cells or tissues was extracted using Trizol reagent (Life Technologies), and reversely transcribed into cDNA. The expression levels of indicated genes were measured by real time PCR on ABI 7700 system using the following primers: forward PNCK-F: 5'-TGACATCTCAGAATCAGCCAAAG-3', reverse PNCK-R 5'-GTGTCCGAGCAAAGTTCTTCC-3'; GAPDH-F: 5'- TGACTTCAACAGCGACACCCA-3', reverse GAPDH-R 5'- CACCCTGTTGCTGTAGCCAAA-3'. PCR was conducted with the following parameters: an initial denaturation step of 30 seconds at 95°C; and 94°C (30 seconds), 58°C (30 seconds), 72°C (50 seconds) in a total 35 cycles with a final extension step at 72°C for 5 min. All samples were normalized to GAPDH and expression fold changes were calculated using 2-ΔΔCt methods.

### Western blotting

Cells were collected, lysed in RIRA lysis buffer, and subject to centrifugation at 12,000 rpm for 10min. The protein concentration was determined using a BCA protein assay kit (Beyotime, Haimen, China). Equal amounts of protein lysates were separated by denaturing sodium dodecyl sulfate-polyacrylamide gels and transferred to nitrocellulose membranes (Bio-Rad Laboratories, Hercules, CA). After blocking in 5% nonfat dry milk, the membranes were incubated with primary antibodies targeting PNCK (1:500, Sigma, USA), PI3K (1:1000, Cell Signaling Technology, USA), AKT (1:1000, Cell Signaling Technology, USA), mTOR (1:1000, Cell Signaling Technology, USA), p-PI3K (1:1000, Abcam, USA), p-AKT (1:2000, Cell Signaling Technology, USA), p-mTOR (1:5000, Cell Signaling Technology, USA) and GAPDH (1:1000, Santa Cruz, USA) at 4℃ overnight. The membranes were rinsed with PBST (PBS with 0.1% Tween-20) three times and incubated with the appropriate secondary antibodies for 1h at room temperature. The protein bands were detected using an enhanced chemiluminescence kit (GE Healthcare, Piscataway, NJ).

### Xenograft model

Female athymic BALB/c mice (4-6 weeks old) were purchased from Shanghai Slac Laboratory Animal Co. Ltd. (Shanghai, China). All procedures for animal studies were approved by Animal Care and Use Committee of Fujian Cancer Hospital. Wild-type or PNCK-knockdown CNE-2 cells were injected subcutaneously into nude mice (n = 10 per group). After 10 days, tumors were measured with calipers once a week for five weeks. Tumor volume was calculated according to the following formula: V = π/6 × L × W^2^ where V= volume (mm^3^), L = length (mm), and W = width (mm) 13. At the end of experiments, mice were euthanized and tumors were excised and weighed. For measurement of bioluminescence imaging density, mice were anesthetized with isoflurane gas and injected intraperitoneally with 150 mg/kg D-luciferin aqueous solution. The images were captured 10 min following injection using an IVIS 50 Imaging system (Caliper Life Sciences, Alameda, CA, USA). Signal intensity was quantified within a region of interest.

### Statistical analysis

All statistical analyses were performed with SPSS 17.0. The data were presented as the mean ± standard deviation (S.D.) of at least three independent experiments. Differences between groups were compared using Student's* t* test or ANOVA. All data were considered significant difference if P values were < 0.05.

## Results

### Up-regulation of PNCK in human NPC

**Figure 1:**
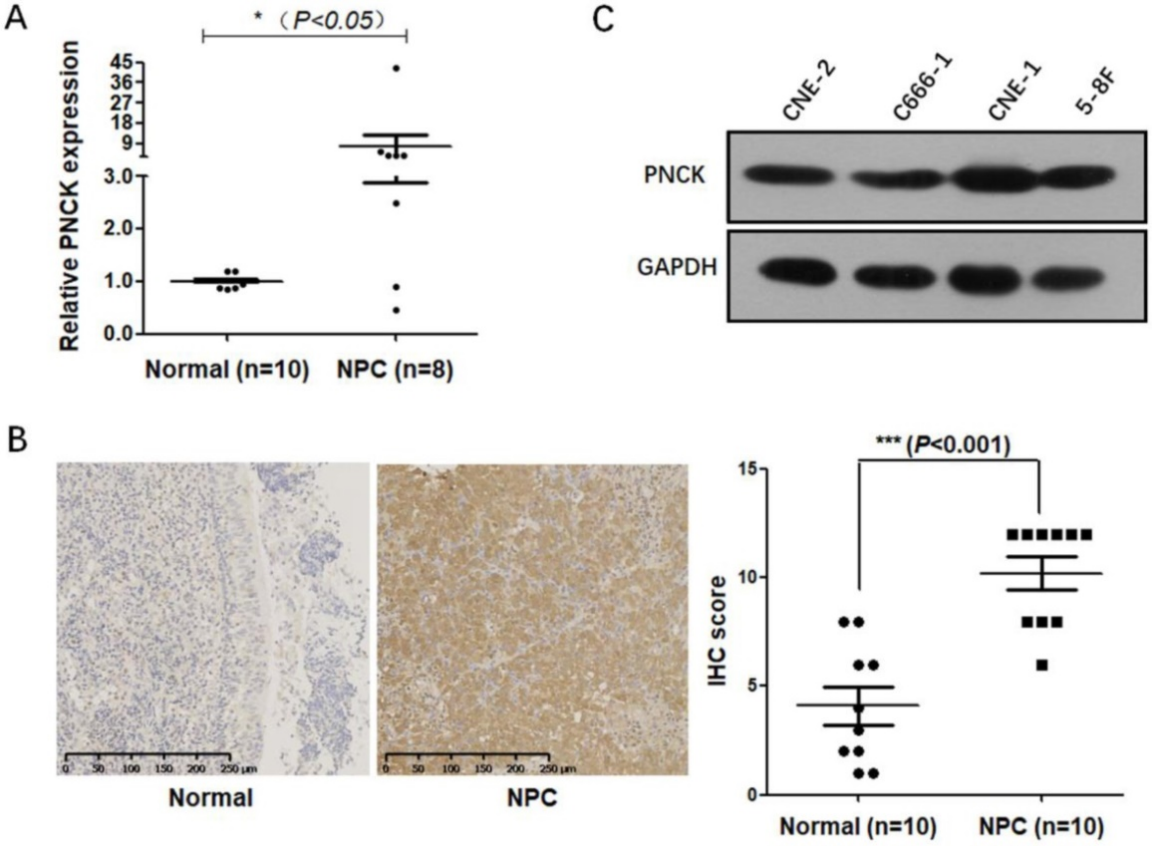
** Up-regulation of PNCK in human NPC.** The expression of PNCK in NPC tumor tissues and the matched adjacent nasopharyngeal tissues was detected by real time PCR (A) and immunohistochemical staining (B). (C) Western blot was performed to detect the expression of PNCK in four NPC cell lines including CNE-2, C666-1, CNE-1 and 5-8F.

Firstly, mRNA and protein levels of PNCK in NPC cancerous and normal tissues were determined by real-time PCR and immunohistochemical staining. The results (Fig 1A and B) showed that the mRNA and protein levels of PNCK were higher in the NPC tissues compared to the normal tissues. Furthermore, Figure 1C showed that PNCK in four NPC cell lines (CNE-2, C666-1, CNE-1 and 5-8F) was detectable, indicating that PNCK is commonly possessed by NPC cells.

### ShRNA-mediated PNCK knockdown inhibited growth and induced apoptosis in NPC cells

**Figure 2:**
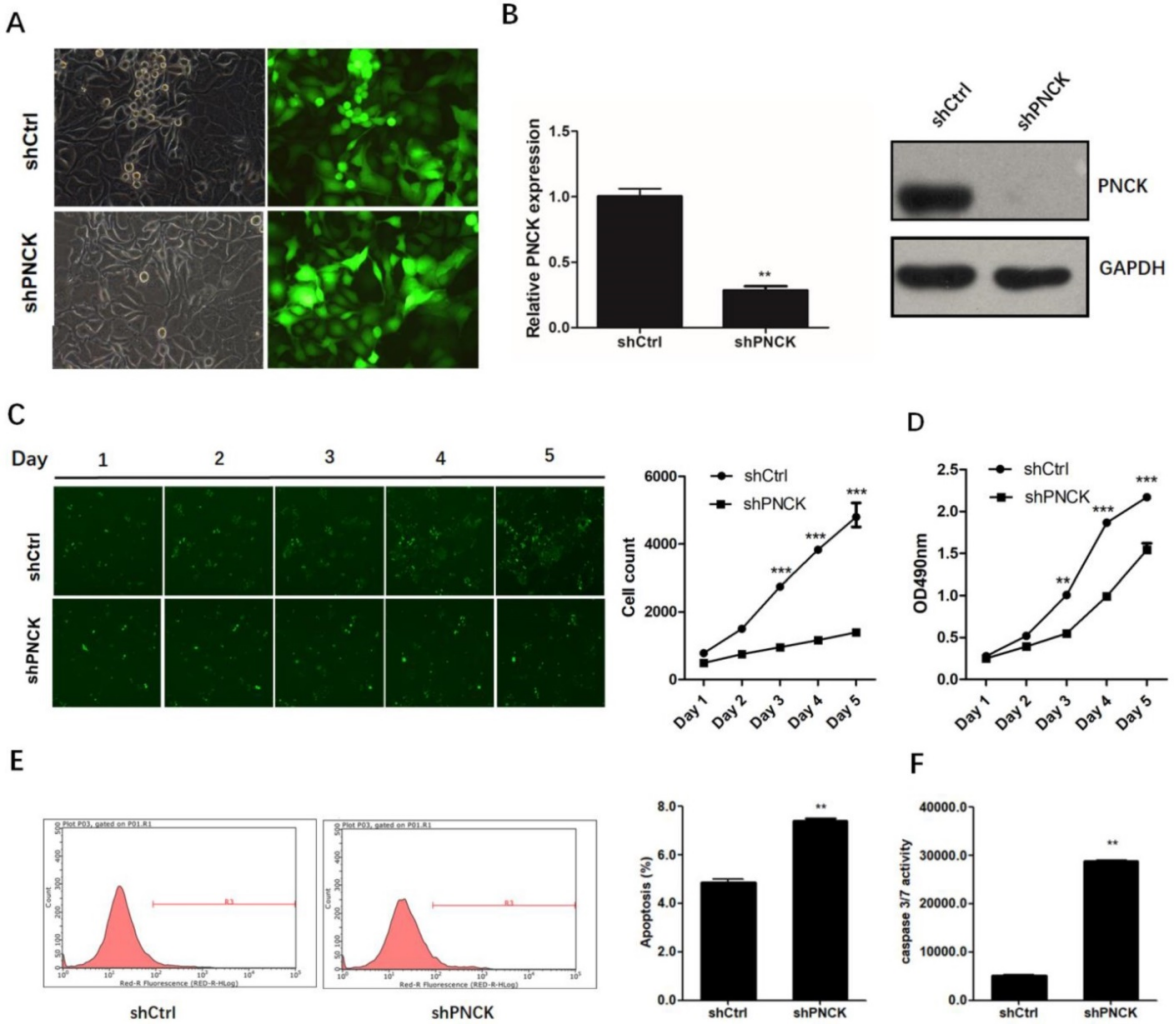
** ShRNA-mediated PNCK knockdown inhibited growth and induced apoptosis in NPC cells.** Human NPC CNE-2 cells were infected with PNCK shRNA using a lentivirus-GFP system (A). After 72 h, silencing of PNCK expression was validated by using real time PCR (B) and western blot analysis (C). Celigo Cell Counting (C) and MTT assays (D) were conducted to measure the effect of PNCK knockdown on cell viability. The effect of PNCK knockdown on cell apoptosis was assessed by determining the apoptotic cell death (E) and caspase 3/7 activity (F). ** P<0.01, *** P<0.001.

To investigate the role of PNCK in the growth of NPC cells, we knocked-down the expression of PNCK by shRNA in CNE-2 cells (Figure 2A) at the mRNA and protein levels as confirmed by real-time PCR and western blot analysis (Figure 2B). The growth and proliferation of PNCK knocked-down cells was inhibited compared to control cells demonstrated by Celigo Cell Counting (Figure 2C) and MTT assays (Figure 2D). Furthermore, the PNCK knockdown could enhance the programmed cell death (apoptosis), as showed in Annexin V-FCM assay (Figure 2E). Moreover, the caspase 3/7 activity in PNCK knockdown tumor cells was elevated in ELISA assay (Figure 2F).

### CRISPR-Cas9-mediated PNCK knockout induced apoptosis in NPC cells

**Figure 3:**
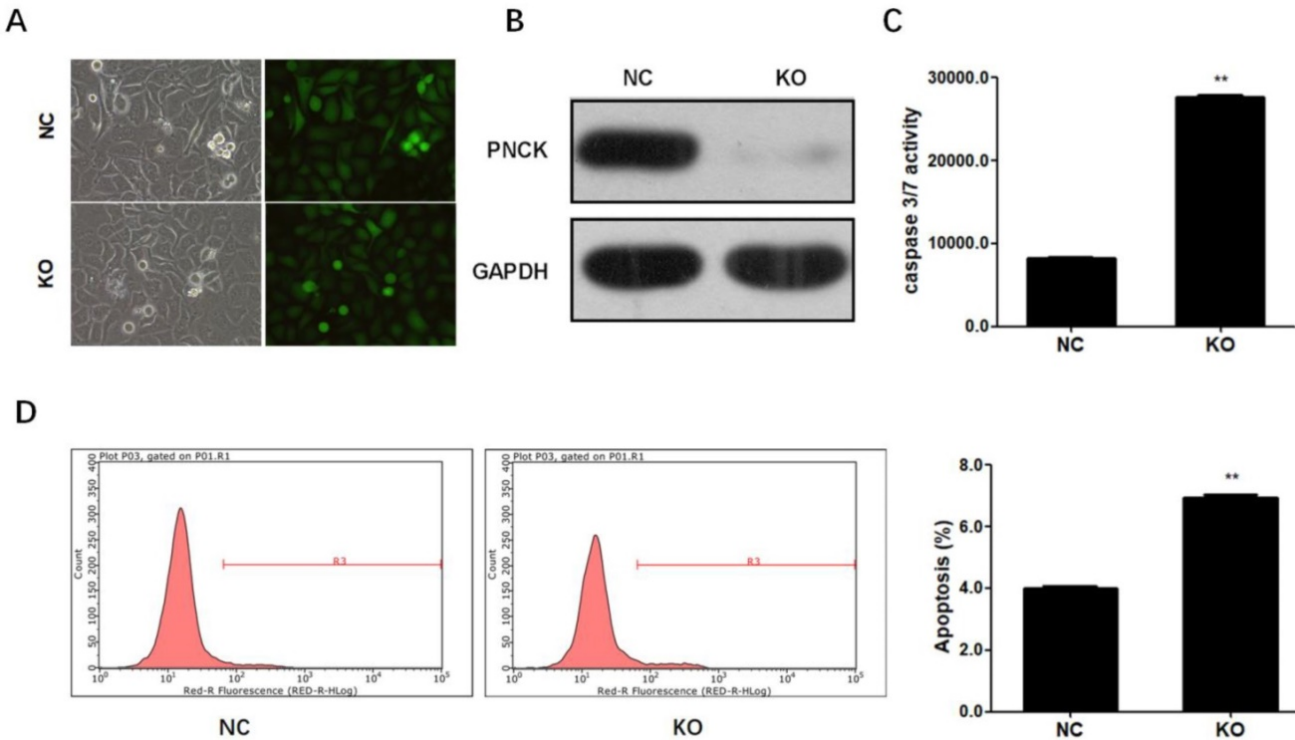
** CRISPR-Cas9-mediated PNCK knockout induced apoptosis in NPC cells.** Human NPC CNE-2 cells were co-transfected with CRISPR-Cas9 and sgRNA against the PNCK (A). After 72 h, silencing of PNCK expression was validated by using western blot analysis (B). The effect of depletion of PNCK on cell apoptosis was assessed by determining the caspase 3/7 activity (C) and apoptotic cell death (D). ** P<0.01.

To completely deplete PNCK, CNE-2 cells were co-transfected with CRISPR-Cas9 and sgRNA against the PNCK (Figure 3A). Western blot analysis showed that PNCK was completely silenced by CRISPR/Cas9-mediated gene knockout (Figure 3B). In addition, ELISA assay showed that PNCK knockout significantly increased the caspase 3/7 activity in CNE-2 cells (Figure 3C). Consistently, flow cytometric analysis revealed an elevated apoptosis in PNCK knockout CNE-2 cells (Figure 3D). Collectively, these data indicated that knockout of PNCK induced apoptosis in NPC cells.

### *In vivo* effect of PNCK knockdown on NPC cell growth

**Figure 4:**
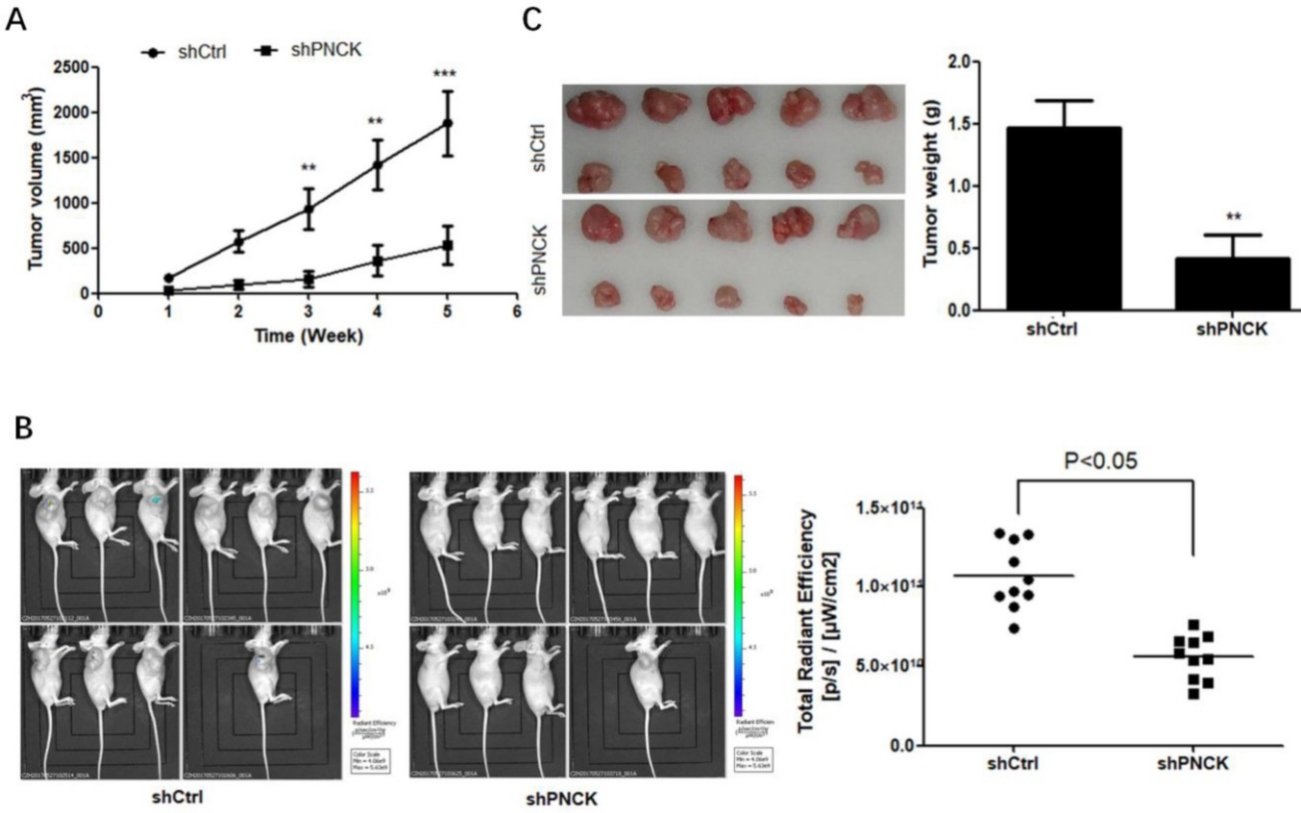
** Depletion of PNCK inhibited tumor growth* in vivo*.** The wild-type or PNCK-knockdown CNE-2 cells were injected subcutaneously into the flank of nude mice. Tumor diameters were measured at a regular interval of one week for up to five weeks, and the tumor volume was calculated (A). (B)* In vivo* bioluminescence imaging of tumors in wild-type or PNCK-knockdown CNE-2 tumor bearing mice. The xenografts were harvested after five weeks. The pictures of the tumors were taken, and the weights of the tumors were analyzed (C). ** P<0.01, *** P<0.001.

To explore if the *in vitro* effect of PNCK-knockdown can be translated *in vivo*, the PNCK knockdown CNE-2 cells were injected subcutaneously into the flank of BABL/c nude mice. After 2 weeks later, the tumor volume in each mouse was measured once a week for consecutive 5 weeks. There was a significant reduction of tumor volume and bioluminescence imaging (BLI) signals in tumors formed by PNCK-knockdown CNE-2 cells compared to those formed by control cells (Figure 4A and B). Similarly, the lack of PNCK significantly inhibited the tumor weight tumors at the end of this study as compared with the tumors formed by wild-type NPC cells (Figure 4C). Collectively, these data indicate that inhibition of PNCK exhibits anti-proliferative effect in *in vivo* model of xenograft nude mice.

### Effects of PNCK depletion on PI3K/AKT/mTOR signaling pathway

Subsequently, genome-wide expression profiling was conducted to determine the differentially expressed genes in the CNE-2 cell line upon PNCK knockdown. Total RNAs were extracted from the wild type or PNCK-knockdown CNE-2 cells and subjected to microarray analysis using Affymetrix GeneChip PrimeView Human Gene Expression Arrays. The purity (A260/280 value) of all RNA samples prepared was >1.95 (Table 1).

**Figure 5:**
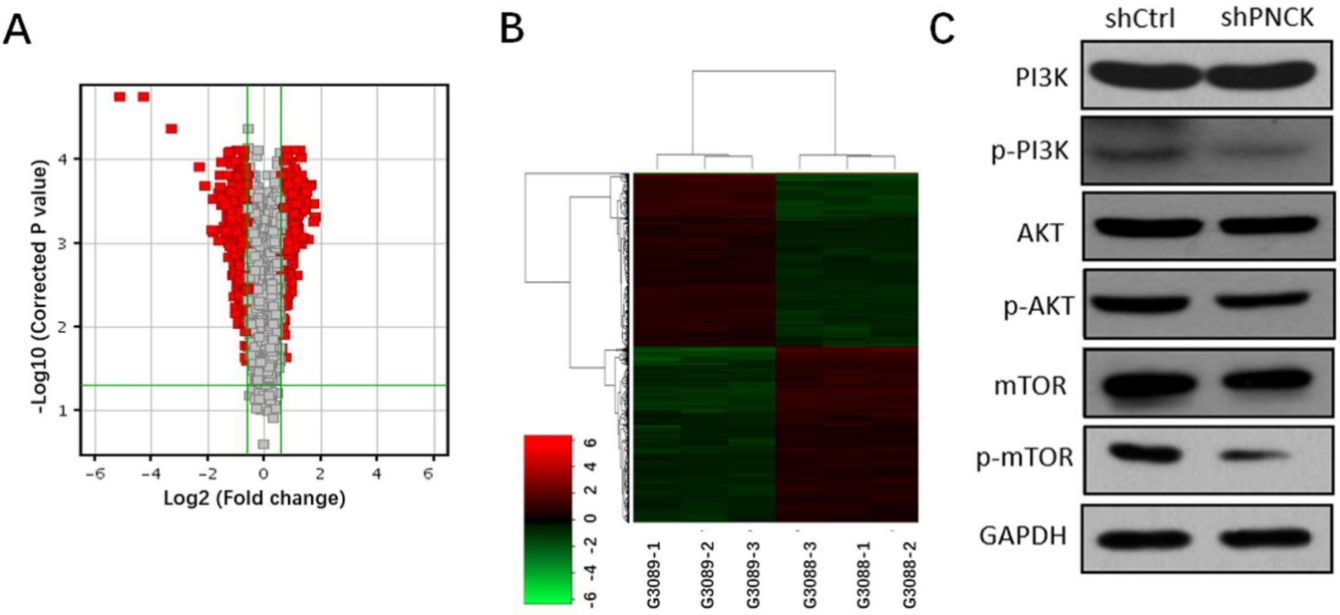
** Effects of PNCK depletion on PI3K/AKT/mTOR signaling pathway.** Volcano plots (A) and cluster analysis (B) in NPC cells after depletion of PNCK. (C) The expression of PI3K, AKT and mTOR and their phosphorylation levels in NPC cells were evaluated by western blot.

Transcriptomic analysis revealed 589 genes were up-regulated and 589 genes were down-regulated (fold change >=1.5, P-value<0.05). Volcano plots and heat maps of these differentially expressed genes visualized a clear distinction between wild type and PNCK knockdown CNE-2 cells (Figure 5A and B). Moreover, pathway analysis using Ingenuity Pathway Analysis (IPA) revealed significant changes of PI3K/AKT/mTOR signaling pathway. Furthermore, western blot analysis showed that interference with PNCK expression reduced the phosphorylation levels of PI3K, AKT and mTOR in CNE-2 cells (Figure 5C). There were no significant changes of total PI3K, AKT and mTOR between control and shPNCK groups. Collectively, these findings reveal that depletion of PNCK inhibits PI3K/AKT/mTOR signaling pathway in NPC cells.

## Discussion

In this study, the high expression of PNCK was detected in NPC tumor tissues and NPC cell lines. Silencing of PNCK inhibited cell proliferation and induced apoptosis *in vitro*. Moreover, animal experiments revealed that PNCK knockdown suppressed tumor growth. In addition, transcriptomic analysis indicated substantial changes in the gene expression and various signaling pathways involved in tumor growth and development. We conclude that PNCK plays a potential oncogenic role in NPCs by promoting NPC cell proliferation and tumor growth, a new insight into understanding the mechanisms of NPC tumorigenesis and exploring the potential therapeutic targets for NPCs.

PNCK, a newly discovered CaM kinase, is elevated in the mouse mammary gland during pregnancy in a subset of epithelial cells 10. Similarly, PNCK is up-regulated in the transformation of human breast cancer, suggesting that PNCK may be involved in mammary development and carcinogenesis 11. In addition, high expression of PNCK is reported in clear cell renal cell carcinoma (ccRCC), which is correlated with shorter overall survival, an independent prognostic factor for survival in ccRCC 14. Recently, studies have revealed that PNCK induces ligand-independent EGFR degradation by probable perturbation of the Hsp90 chaperone complex, representing an attractive target in EGFR-regulated oncogenesis 15-17. Additionally, ectopic expression of PNCK causes trastuzumab resistance in HER-2 amplified breast cancer via PTEN-mediated process, suggesting that inhibition of PNCK may be a novel strategy to overcome drug resistance 18.

Gene expression profiling, pathway analysis and IPA analysis by comparison of with PNCK-depleted NPC cells with its wild-type cells revealed that hundreds of genes were significantly altered after PNCK knockdown, especially the signaling pathways (i.e., PI3K/AKT/mTOR, IL-1 and integrin signaling) and biological events (i.e., cell deaths and survival, apoptosis, cancer pathogenesis, cellular development). The PI3K/AKT/mTOR pathway is a survival pathway constitutively activated in many types of cancer. This pathway is considered as an attractive therapeutic target for cancer treatment because it serves as a convergence point, controlling the multiple biological processes that contribute to the initiation and progression of cancer 22,23. It has been reported that blocking PI3K/AKT signaling attenuates metastasis of NPC cells by inducing mesenchymal-epithelial reverting transition 24. Moreover, genetic variations in the PI3K/AKT/mTOR pathway are suggested to be involved in the distant metastasis in NPC patients 25. Accordingly, many studies have performed the preclinical evaluation of small-molecule inhibitors that target PI3K-mTOR as a novel therapeutic drug in NPC 26-28. Consistently, our study validated that interference with PNCK expression reduced the phosphorylation levels of PI3K, AKT and mTOR, which contributed to the inhibition of PI3K/AKT/mTOR signaling pathway in NPC cells.

However, there are several limitations of this study, such as small sample size and lack of follow-up data that could have provided possible association between PNCK expression and clinicopathologic characteristics such as TNM stage, age, gender and survival. Although we found that PI3K/AKT/mTOR pathway is regulated by PNCK, the detailed regulation mechanisms are not understood. Therefore, a further investigation into the underlying molecular interaction between PNCK and PI3K/AKT/mTOR in NPC is needed.

In conclusion, the present study, for the first time, demonstrated the up-regulation of PNCK in NPC. Interference with PNCK expression exhibited an anti-oncogenic role in NPC cells both* in vitro* and *in vivo*. Moreover, transcriptomic analysis revealed that PNCK depletion induced substantial changes of gene expression and transduction signals. These findings indicate that PNCK might be a potential therapeutic target of NPC.

## Figures and Tables

**Table 1:** Preparation of RNAs for microarray analysis*.

Sample number	Name	Thermo NanoDrop 2000	
		Concentration(ng/μL)	A260/A280
G3088-1	NC	491.8	1.96
G3088-2	NC	505.6	1.97
G3088-3	NC	522.8	2.02
G3089-1	KD	491.1	1.97
G3089-2	KD	485.5	1.97
G3089-3	KD	500.1	1.97

*NC: control wild-type CNE-2 cells; KD: PNCK knockdown CNE-2 cells. A total of six samples were subjected to microarray analysis with three samples in each group.
